# DNA methylation signature of passive smoke exposure is less pronounced than active smoking: The *Understanding Society* study

**DOI:** 10.1016/j.envres.2020.109971

**Published:** 2020-11

**Authors:** Paige M. Hulls, Frank de Vocht, Yanchun Bao, Caroline L. Relton, Richard M. Martin, Rebecca C. Richmond

**Affiliations:** a Population Health Sciences, Bristol Medical School, University of Bristol, BS8 2BN, UK; b MRC Integrative Epidemiology Unit at University of Bristol, BS8 2BN, UK; c NIHR Applied Research Collaboration West (NIHR ARC West), Bristol, BS1 2NT, UK; d Institute for Social and Economic Research, University of Essex, CO4 3SQ, UK; e NIHR Bristol Biomedical Research Centre at the University Hospitals Bristol NHS Foundation Trust and the University of Bristol, BS8 2BN, UK

**Keywords:** DNA methylation, EWAS, Understanding society, Passive smoke exposure

## Abstract

**Introduction:**

The extent of the biological impact of passive smoke exposure is unclear. We sought to investigate the association between passive smoke exposure and DNA methylation, which could serve as a biomarker of health risk.

**Materials and methods:**

We derived passive smoke exposure from self-reported questionnaire data among smoking and non-smoking partners of participants enrolled in the UK Household Longitudinal Study ‘*Understanding Society’* (n=769). We performed an epigenome-wide association study (EWAS) of passive smoke exposure with DNA methylation in peripheral blood measured using the Illumina Infinium Methylation EPIC array.

**Results:**

No CpG sites surpassed the epigenome-wide significance threshold of p<5.97 × 10^−8^ in relation to partner smoking, compared with 10 CpG sites identified in relation to own smoking. However, 10 CpG sites surpassed a less stringent threshold of p<1 × 10^−5^ in a model of partner smoking adjusted for own smoking (model 1), 7 CpG sites in a model of partner smoking restricted to non-smokers (model 2) and 16 CpGs in a model restricted to regular smokers (model 3). In addition, there was evidence for an interaction between own smoking status and partners’ smoking status on DNA methylation levels at the majority of CpG sites identified in models 2 and 3. There was a clear lack of enrichment for previously identified smoking signals in the EWAS of passive smoke exposure compared with the EWAS of own smoking.

**Conclusion:**

The DNA methylation signature associated with passive smoke exposure is much less pronounced than that of own smoking, with no positive findings for ‘expected’ signals. It is unlikely that changes to DNA methylation serve as an important mechanism underlying the health risks of passive smoke exposure.

## Highlights

• Investigated the association between blood-based genome-wide DNA methylation and SHS exposure.
• Data were drawn from smoking and non-smoking partners of participants in the *Understanding Society* study.
• No CpG sites surpassed the epigenome-wide significance threshold in relation to partner smoking.
• The DNA methylation signature associated with SHS exposure is much less pronounced than that of own smoking.

## 1 Introduction

Passive smoke (PS), is the exposure to “*second- or third-hand smoke by breathing ambient air containing toxic substances resulting from the combustion of tobacco products after birth or the exposure to utero to maternal blood contaminated of tobacco smoking products*” (Gibson et al., 2013). In spite of progress in tobacco control, a large proportion of the world's population remain exposed to second-hand smoke: in 2011 this was estimated to be 40% of children, 33% of male non-smokers and 35% of female non-smokers (Öberg et al., 2011). Second-hand smoke (SHS) contains at least 5,000 chemicals, including over 70 that are known to be carcinogenic (Cancer Research UK, 2018). While the chemical constituents differ from primary tobacco smoke inhalation, and although the levels of exposure are much less than personal smoking, the 2006 US Surgeon General's report concluded that there is also no risk-free level of SHS exposure (Department of Health, 2014; Department of Health, 2010). In addition, the risks of passive smoke exposure do not stop at second-hand smoke, but may also come from exposure to third-hand smoke, which is the residue of tobacco smoke that persists long after a cigarette has been extinguished (Protano and Vitali, 2011). These conclusions about the damaging impact of PS on adult health draw on reported associations with increased risks of lung cancer, and respiratory and cardiovascular diseases (ASH, 2014). Furthermore, there is some evidence that children exposed to PS can be more likely to take up smoking themselves (Leonardi-Bee et al., 2011).

In contrast, several large-scale studies have not supported a causal relationship between PS exposure and tobacco-related mortality (Enstrom et al., 2003). Such contradictory results could potentially reflect misclassification and measurement error, especially as PS is often measured via self-report (Davey-Smith, 2003). Cotinine (measured in urine, blood or sputum) is a metabolite of nicotine which has been widely used as a biomarker for personal smoke exposure, although it may also serve as a marker of PS exposure (Benowitz, 1996; Etzel, 1990). A previous study conducted using data from the Avon Longitudinal Study of Parents and Children (ALSPAC) showed low levels of cotinine in non-smoking women whose partners smoked (Taylor et al., 2014), indicating a potentially negligible biological effect of PS from partners. However, because of the short half-life of cotinine it may not provide an accurate representation of long-term PS exposure and related effects (Benowitz and Jacob, 1993).

DNA methylation is a type of epigenetic process characterised by the addition of methyl groups to sites in the DNA known as “CpGs” (cytosine-phosphate-guanine). It is altered in response to various environmental and biological factors and in recent epigenome-wide association studies (EWAS), cigarette smoking has been associated with long-term changes in DNA methylation (Breitling et al., 2011; Joehanes et al., 2016). For example, peripheral blood methylation at sites in the DNA annotated to genes such as *AHRR* (aryl hydrocarbon receptor repressor) has been shown to determine previous smoke exposure with much higher accuracy than cotinine levels (Zhang et al., 2016) and has been shown to predict future lung cancer risk (Zhang et al., 2015).

Whilst peripheral blood DNA methylation changes have been identified in relation to intra-uterine exposure to maternal smoking (Joubert et al., 2016; Richmond et al., 2015), there has been only one study investigating the association between postnatal PS exposure and peripheral blood DNA methylation variation, which has evaluated methylation only at a limited number of CpG sites (Reynolds et al., 2017).

This study aimed to: i) investigate the association between blood-based genome-wide DNA methylation and PS exposure, based on questionnaire derived information on partners' smoking behaviour and ii) compare the DNAm signature for PS exposure with that associated with the participants’ own smoking behaviour (active smoking).

## 2 Materials and methods

### 2.1 Study description

The UK Household Longitudinal Study (UKHLS) (Knies, 2017), also known as *Understanding Society* (University of Essex, 1991–2009), is an ongoing longitudinal panel survey of 40,000 UK households from England, Scotland, Wales and Northern Ireland (Buck and McFall 2011).The survey started in 2009 and collects information about people's health, behaviours, attitudes and social and economic circumstances. Further details on *Understanding Society* sample methodology has been published elsewhere (Lynn, 2009).

### 2.2 Measures

Annual interviews have collected sociodemographic information since 1991. Biomedical measures and blood samples were collected at a nurse visit in the participants’ homes between 2010 and 2012. The eligibility criteria for collecting blood samples were: participation in the previous main interview in England; age 16 and over; living in England, Wales or Scotland; not pregnant; and other conditions covered in the user guide (Benzeval et al., 2017).

#### 2.2.1 Genome-wide quantification of peripheral blood DNA methylation variation

DNA was extracted from the blood samples of 1193 eligible individuals aged 28 to 98 who had consented to both blood sampling and genetic analysis, had participated in all annual interviews between 1999 (BHPS wave 9) and 2011–2013 (*Understanding Society* wave 3), and whose time between blood sample collection and processing did not exceed three days. Eligibility requirements for genetic analyses meant that the samples for DNA methylation measurement were restricted to participants of white ethnicity. 500ng of DNA from each sample was bisulphite converted using the EZ-96 DNA methylation-Gold Kit (Zymo Research, CA, USA). DNA methylation was quantified using the Illumina Infinium HumanMethylationEPIC BeadChip (Illumina Inc., CA, USA) run on an Illumina iScan System (Illumina, CA, USA) using the manufacturer's standard protocol. Samples were randomly assigned to chips and plates to minimise batch effects. A fully methylated control (CpG Methylated HeLa Genomic DNA; New England BioLabs, MA, USA) was included in a random position on each plate to facilitate tracking, resolve experimental inconsistencies and confirm data quality (University of Essex, 1991–2009).

#### 2.2.2 DNA data pre-processing

Using the “bigmelon” package in R (Gorrie-Stone et al., 2018), raw signal intensities were imported from idats and converted into beta values. Data were processed through a standard pipeline and included the following steps: outlier detection, confirmation of complete bisulphite conversion, and estimation of age from the data (Horvath, 2013). Data were normalised using the “dasen” function from the “watermelon” package in R software (Pidsley et al., 2013). Samples that were dramatically altered as a result of normalisation were excluded by assessing the difference between normalised and raw data and removing those with a root mean square and standard deviation > 0.005. Samples where >1% of sites or sites where >1% of samples had a p-value for detection >0.05 were also excluded. DNA methylation sites with a bead count <3 were excluded. The data were then re-normalised with the “dasen” function. The final dataset included 857,071 DNA methylation (CpG) sites of 1175 individuals. For the current analysis, CpG sites residing on the X or Y chromosome were excluded, as were SNP and control probes, leaving 837,487 CpG sites for analysis.

#### 2.2.3 Technical variation

Batch effects were accounted for by adjusting for the batch numbers of the blood samples and batch number of the samples in the lab. Blood cell composition estimates were calculated using the Houseman reference based algorithm implemented in the “estimateCellCounts” function packaged “minfi” (Houseman et al., 2012; Aryee et al., 2014) and included as covariates in the statistical models.

#### 2.2.4 Exposure assessment

The *Understanding Society* questionnaires are completed via interviews with members of each household that are over the age of ten. Questionnaire data for the 1175 individuals with DNA methylation data were obtained from annual surveys conducted as part of BHPS (waves 9–18; 1999–2009) and *Understanding Society* (wave2; 2010–2012). In wave 2 of *Understanding Society*, participants were asked about their smoking history and whether they had ever smoked a cigarette, a cigar or a pipe. Participants who responded with ‘no’ were classed as “never smokers”. Participants who responded with ‘yes’ and who also reported that they had ever smoked cigarettes, a cigar or a pipe regularly (at least one per day) were classed as “regular smokers”. To maximise contrast, somewhat ambiguous groups, including participants who responded with ‘yes’ but who reported that they had not smoked cigarettes, a cigar or a pipe regularly were excluded from the analysis. This was to exclude those individuals with only a limited smoking history (i.e. less than one per day). We ran an additional analysis whereby we assessed partners' regular smoking among participants who reported being never smokers themselves.

#### 2.2.5 Covariates

All models were adjusted for age at nurse visit, sex, six estimated cellular composition variables (B cells, CD8 T cells, CD4 T cells, monocytes, granulocytes, natural killer T cells), two batch variables (blood processing day and batch number) and surrogate variable analysis derived from the data using “meffil” (Min et al., 2018). Inclusion of these modelled factors in the epigenome-wide association analyses was justified given their substantial loadings on the top principal components generated from the DNA methylation data using singular value decomposition analysis in “ChAMP” (Tian et al., 2017) (***Supplementary File 1***). Surrogate variable analysis was used to capture large-scale effects of unmodelled factors in order to overcome sources of heterogeneity in the EWAS and to increase the biological accuracy and reproducibility of analyses (Leek and Storey, 2007).

### 2.3 Statistical analyses

#### 2.3.1 Epigenome-wide association analysis

DNA methylation variation (as the outcome variable) was analysed in relation to PS (as the exposure variable) using multivariable regression in an epigenome-wide association study (EWAS) approach, in the R package “meffil” designed for quality control, normalisation and EWAS of large samples of Illumina Methylation BeadChip microarrays (Min et al., 2018). The main EWAS analysis was for ever regular partner smoking versus never partner smoking, with adjustment for participants' own smoking status (never or regular smoker) (model 1). We also conducted a subgroup analysis for regular partner smoking among never smokers only (model 2) and compared this to regular partner smoking among regular smokers (model 3). Based on this subgroup analysis, we assessed whether there was an interaction between own smoking status and partner's smoking status on DNA methylation variation, using a Cochran's Q test.

Results of models 1–3 were directly compared with an EWAS analysis for participants' own smoking status (ever regular versus never) with adjustment for partners’ ever smoking status. As own smoking has been robustly associated with changes in DNA methylation previously (Joehanes et al., 2016), this analysis served as a positive control (model 4).

We further investigated CpG sites that reached a Bonferroni-significance threshold of P<5.97 × 10^−8^, as well as a less stringent threshold of P<1.00 × 10^−5^, in order to assess concordance of DNA methylation signals across the models. While we could not make claims about epigenome-wide significance for those CpGs surpassing the less stringent threshold, this was used to assess concordance of DNA methylation signals across the models and in relation to other published EWAS. Given previous evidence of an association between SHS exposure and DNA methylation at cg05575921 (*AHRR*) (Reynolds et al., 2017), we also specifically investigated the strength of associations at this CpG site in all of the models.

Finally, we investigated overlap of the CpG sites identified with those previously reported in the published literature by: 1) performing a search of the top CpG sites from the EWAS performed in two publicly available repositories: the EWAS Catalog (MRC-IEU University of Bristol, 2018) and EWAS Atlas (BIG Data Center, 2019), and 2) assessing whether there was any evidence for enrichment of previously identified CpG sites related to own smoking status from a large meta-analysis (Joehanes et al., 2016) in our EWAS of PS exposure. We assessed the degree of inflation of association signal (lambda value) for these CpG sites compared with that seen genome-wide across the samples and performed a Wilcoxon rank sum test to assess enrichment.

Analysis was performed using Stata (version 15) and R (version 3.5.1).

## 3 Results

### 3.1 Study characteristics

**Fig. 1:**
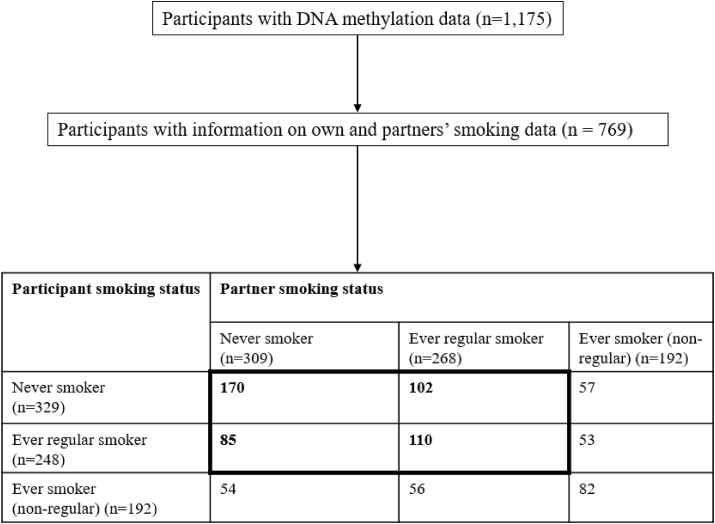
Flow diagram of participants stratified by smoking status in Epigenome-Wide Association Analysis (EWAS) models.

Compared with those individuals aged 28 to 98 who were originally part of the BHPS cohort and who had questionnaire data from wave 2 of *Understanding Society* (n=8551), those with epigenetic data were slightly older on average and were more likely to be female, to have been a regular smoker and to have had a partner who smoked (Table 1). Of the 1175 individuals with epigenetic data, there were 769 participants whose partners reported their own smoking status. 35% of the 769 participants had partners who were regular smokers (268 participants). Of these 268, 41% (110) had previously smoked themselves, either regularly or non-regularly. The mean age of all 769 participants was 56.5 years (standard deviation (SD) ± 14.5), whilst for participants with partners that smoked regularly the mean age was 58.4 years (SD ± 12.2). 52.1% of the participants were female and the total sample had a mean body mass index (BMI) of 28.3 (SD ± 5.3). For participants with partners who had smoked regularly, the percentage of women (64.2%) was higher than for participants with partners who had never smoked (41.5%). Participants with partners who had smoked regularly had a BMI of 28.6 (SD ± 5.4) in comparison to a BMI of 27.9 (SD ± 4.6) for participants with partners who had never smoked. Within the sample (n=769), 19.9% self-reported having a degree as their highest qualification, 24.9% had a GCSE, and 13.6% reported having no qualifications (Table 2). Fig. 1 shows the classification of participants and their partners in each EWAS model.

**Table 1:** Representativeness of participants with epigenetic data in *Understanding Society*.

Demographics	Participants with epigenetic data (n=1175) [SD]	BHPS Participants at wave 2 of Understanding Society^a^ (n=8551) [SD]	P-value for difference
Age (n=1175; n=8551)	56.5 (15.0)	53.0 (15.9)	<0.0001
Sex (n=1175; n=8551) [males/females]	41.7%/58.4%	46.2%/53.9%	0.004
BMI (kg/m^2^) (n=1142; n=2802)	28.2 (5.2)	28.5 (5.5)	0.093
Educational Attainment (n=1168; n=8458) [degree/other higher degree/A-level/GCSE/ other qual/no qual]	18.1 % 11.1 % 19.1 % 24.3 % 10.0 % 17.4 %	17.7 % 10.3 % 21.0 % 21.7 % 10.3 % 19.0 %	0.203
Participant ever smoker (n=1170; n=8274) [yes/no]	57.4%/42.6%	42.4%/57.6%	0.908
Participant regular smoker (n=861; n=5696) [yes/no]	42.2%/57.8%	38.4%/61.6%	0.036
Partner ever smoker (n = 769; n=5120) [yes/no]	59.8%/40.1%	55.0%/45.0%	0.014
Partner regular smoker (n=577; n=3785) [yes/no]	46.4%/53.6%	39.2%/60.8%	0.001

a Participants aged 28–98 years without epigenetic data.

**Table 2:** Descriptive characteristics of participants by partners smoking status.

Demographics (separated by participant's own smoking status)	Participants with partners who have smoked regularly (n=268)	Participants with partners who have never smoked (n=309)
**Age**	56.1 (SD ± 13.8)	54.8 (SD ± 14.2)
*Participant regular smoker (n=110; n=85)*	58.4 (SD ± 12.9)	54.8 (SD ± 14.2)
*Participant non-regular smoker (n=56; n=54)*	57.9 (SD ± 12.0)	53.1 (SD ± 13.7)
*Participant never smoker (n=102; n = 170)*	59.0 (SD ± 12.1)	53.1 (SD ± 14.0)
**Sex**	35.8% males/64.2% females	58.5% males/41.5% females
*Participant regular smoker (n=110; n=85)*	45.8% males/54.1% females	73.4% males/26.6% females
*Participant non-regular smoker (n=56; n=54)*	30.36% males/69.64% females	70.37% males/29.63% females
*Participant never smoker (n=102; n = 170)*	29.6% males/70.4% females	44.5% males/55.5% females
**BMI (kg/m**^**2**^**)**	28.6 (SD ± 5.4)	27.91 (SD ± 4.6)
*Participant regular smoker (n=110; n=85)*	29.5 (SD ± 5.6)	29.05 (SD ± 4.9)
*Participant non-regular smoker (n=56; n=54)*	28.0 (SD ± 5.1)	27.48 (SD ± 4.4)
*Participant never smoker (n=102; n = 170)*	28.6 (SD ± 5.5)	27.47 (SD ± 4.4)
**Educational Attainment**	Degree: 13.3%	Degree: 25.6%
	Other higher degree: 12.9%	Other higher degree: 10.7%
	A-level: 19.2%	A-level: 21.7%
	GCSE: 24.7%	GCSE: 24.9%
	Other qual: 11.8%	Other qual: 7.1%
	No qual: 18.1%	No qual: 9.7%
Participant regular smoker (n=110; n=85)	Degree: 12.7%	Degree: 17.6%
	Other higher degree: 11.8%	Other higher degree: 9.4%
	A-level: 21.8%	A-level: 22.4%
	GCSE: 21.8%	GCSE: 27.1%
	Other qual: 14.5%	Other qual: 9.4%
	No qual: 17 .3%	No qual: 14.1%
Participant non-regular smoker (n=56; n=54)	Degree: 12.5%	Degree: 22.2%
	Other higher degree: 10.7%	Other higher degree: 9.3%
	A-level: 16.1%	A-level: 29.6%
	GCSE: 19.6%	GCSE: 24.1%
	Other qual: 19.6%	Other qual: 7.4%
	No qual: 21.4%	No qual: 7.4%
Participant never smoker (n=102; n=170)	Degree: 14.4%	Degree: 29.9%
	Other higher degree: 15.4%	Other higher degree: 11.9%
	A-level: 17.3%	A-level: 19.2%
	GCSE: 30.8%	GCSE: 25.1%
	Other qual: 4.8%	Other qual: 5.9%
	No qual: 17.3%	No qual: 8.4%

n = number; SD = standard deviation; BMI = body mass index.

*The first sample size is participants with partners who smoke, and the second sample size is participants with partners who don't smoke.

### 3.2 Epigenome-wide association study

Results for all CpG sites with a p-value <1 × 10^−5^ from the unrestricted model of partner smoking and the models of partner smoking restricted to non-smokers and smokers are presented in Table 3. Results from the EWAS for own smoking are shown in the ***Supplementary File 2***. Manhattan and QQ plots are shown in ***Supplementary File 3****.*

**Table 3:** Top CpG sites found in Epigenome-Wide Association Study Analysis of Passive Smoking.

Model	N	CpG Site	Position	Chromosome	Gene.symbol	Coef.sva	Se.sva	P.sva	CIL	CIU
Regular Partner Smoking	467	cg04992673	92848714	chr7	HEPACAM2	−0.017	0.003	6.85E-07	−0.010	−0.024
Regular Partner Smoking	467	cg05297322	14105812	chr1	PRDM2	0.009	0.002	9.09E-07	0.014	0.006
Regular Partner Smoking	467	cg26015967	46644841	chr15		−0.012	0.003	2.18E-06	−0.007	−0.017
Regular Partner Smoking	467	cg11549025	75994082	chr7		0.008	0.002	7.40E-06	0.011	0.004
Regular Partner Smoking	467	cg13249774	68121506	chr11	LRP5	−0.012	0.003	9.28E-06	−0.007	−0.018
Regular Partner Smoking	467	cg23410415	14287748	chr6		0.0115	0.0037	7.64E-06	0.016	0.007
Regular Partner Smoking	467	cg18866792	217152056	chr2	MARCH4	−0.015	0.003	3.63E-06	−0.009	−0.021
Regular Partner Smoking	467	cg17672850	40206354	chr17		0.012	0.003	2.34E-06	0.017	0.007
Regular Partner Smoking	467	cg04386216	70130966	chr12	RAB3IP; LOC101928002	−0.014	0.003	8.07E-06	−0.008	−0.020
Regular Partner Smoking	467	cg03796580	80333624	chr17		−0.025	0.005	8.78E-06	−0.014	−0.0361
Regular Partner Smoking (non-smokers)	272	cg19410143	17485612	chr22	GAB4	0.015	0.003	2.16E-06	0.022	0.009
Regular Partner Smoking (non-smokers)	272	cg26874015	134437911	chr10	INPP54	−0.006	0.001	2.28E-06	−0.003	−0.008
Regular Partner Smoking (non-smokers)	272	cg18343108	36997710	chr17	C17orf98	0.039	0.008	2.37E-06	0.054	0.023
Regular Partner Smoking (non-smokers)	272	cg04990241	132147072	chr2		−0.012	0.003	7.88E-06	−0.007	−0.017
Regular Partner Smoking (non-smokers)	272	cg24302327	194311571	chr3	TMEM44;	0.012	0.002	2.91E-06	0.016	0.007
Regular Partner Smoking (non-smokers)	272	cg14795069	158956916	chr7		0.015	0.003	8.46E-06	0.021	0.009
Regular Partner Smoking (non-smokers)	272	cg24445972	133261156	chr7	EXOC4	0.028	0.006	9.61E-06	0.040	0.016
Regular Partner Smoking (smokers)		cg03815796	85260295	chr15	SEC11A	0.025	0.004	9.03E-08	0.029	0.014
Regular Partner Smoking (smokers)		cg25050076	21125547	chr2		−0.018	0.003	6.12E-07	−0.011	−0.025
Regular Partner Smoking (smokers)		cg01006943	126842035	chr12		−0.018	0.004	3.07E-06	−0.011	−0.026
Regular Partner Smoking (smokers)		cg12153072	99481686	chr13	DOCK9	0.022	0.005	1.21E-06	0.014	0.006
Regular Partner Smoking (smokers)		cg14597637	128365555	chr9	MAPKAP1	−0.018	0.004	8.75E-06	−0.010	−0.026
Regular Partner Smoking (smokers)		cg07136054	74486177	chr4	RASSF6	0.009	0.002	1.26E-06	0.014	0.006
Regular Partner Smoking (smokers)		cg11986310	65728298	chr11	SART1	−0.018	0.004	5.72E-06	−0.010	−0.025
Regular Partner Smoking (smokers)		cg04359639	7199917	chr1	CAMTA1	−0.034	0.008	9.21E-06	−0.019	−0.049
Regular Partner Smoking (smokers)		cg08456247	56410143	chr2	CCDC85A	0.022	0.005	4.76E-06	0.032	0.013
Regular Partner Smoking (smokers)		cg21735668	67072353	chr15	SMAD6	0.016	0.003	8.06E-06	0.022	0.009
Regular Partner Smoking (smokers)		cg15003393	22008626	chr1	USP48	0.019	0.004	9.95E-06	0.027	0.011
RegularPartner Smoking (smokers)		cg22512634	19789840	chr1	CAPZB	−0.009	0.002	7.28E-06	−0.006	−0.014
Regular Partner Smoking (smokers)		cg11724883	236785782	chr1		0.024	0.005	8.57E-06	0.034	0.014
Regular Partner Smoking (smokers)		cg01131241	54166783	chr10		0.014	0.003	7.92E-06	0.019	0.008
Regular Partner Smoking (smokers)		cg15163417	61119298	chr17	TANC2	0.014	0.003	7.83E-06	0.019	0.008
Regular Partner Smoking (smokers)		cg24770161	25497323	chr13	CENPJ	−0.006	0.001	6.41E-06	−0.004	−0.009

n = number; coef.sva = coefficient.sva; se.sva = standard error.sva; p.sva = p value from analysis adjusted for surrogate variables; CIL = confidence interval lower limit; CIU = confidence interval upper limit.

In epigenome-wide association analysis of PS exposure, no CpG sites surpassed the epigenome-wide significance threshold of p<5.97 × 10^−8^. 10 CpG sites surpassed a less stringent threshold of p<1 × 10^−5^ in the unrestricted model of partner smoking adjusted for own smoking (model 1), 7 CpG sites in the model of partner smoking restricted to non-smokers (model 2) and 16 in the model of partner smoking restricted to regular smokers (model 3). In the EWAS for own smoking, 10 CpG sites surpassed p<5.97 × 10^−8^ and 30 CpG sites surpassed p<1.00 × 10^−5^ (model 4). Models 1 and 4 had equivalent power since the models included both participants' own smoking status and partners’ ever smoking status, with similar numbers of participants and partners reporting to be regular smokers, along with the same covariates.

**Fig. 2:**
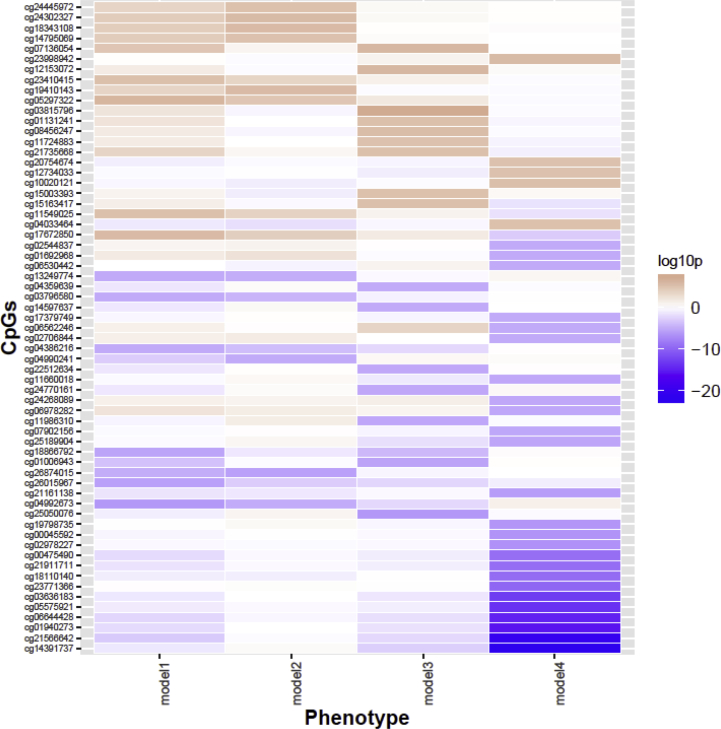
A heatmap to illustrate the direction and strength of association between all investigated passive smoking exposures and DNA methylation. Legend: Plotted CpGs are the top 50 CpGs with the smallest p-values in the passive smoking exposure single-site.

While the sites identified with stratification for smoking (models 2 and 3) exhibited similar levels of DNA methylation in main model (model 1), there were distinct differences in methylation levels found in response to PS exposure among non-smokers and regular smokers (model 2 vs model 3) (Fig. 2). Whereas the majority of the CpGs related to own smoking at p<1 × 10^−5^ were hypo-methylated in relation to own smoking, CpGs related to PS at p<1 × 10^−5^ were more likely to exhibit hyper-methylation (40% hypo-methylated in model 1; 29% in model 2; 43% in model 3). For the majority of the 23 CpG sites identified at p<1 × 10^−5^ in models 2 and 3, there was evidence for an interaction between own smoking status and partners’ smoking status on DNA methylation levels (***Supplementary File 6).*** For example, the top site identified in model 3 (cg03815796, *SEC11A*) exhibited increased methylation in relation to PS exposure among regular smokers (2.2%, 95%CI 1.4%, 2.9%, p=9.03 × 10^−8^), but there was limited evidence for an association with PS exposure among non-smokers (−0.3%, 95%CI -0.9%, 0.3%, p=0.292) (Q statistic = 26.55, p=2.57 × 10^−7^).

There was limited overlap between the sites identified in relation to own smoking (model 4) compared with those identified in relation to partner smoking (models 1–3) (Fig. 2). For some of the sites exhibiting hypomethylation in relation to own smoking, there was some evidence for differential methylation in relation to partner smoking (e.g., at cg21566642: 1.8%, 95% CI -2.9%, −0.8%, p = 8.6 × 10^−4^; and cg06644428: 1.3%, 95% CI -2.2%, −0.4%, p=5.0 × 10^−3^ in model 1). However, the associations were attenuated when own smokers were excluded from the analysis of partner smoking (cg21566642: 0.3%, 95% CI -1.3%, 0.8%, p = 0.63; and cg06644428: 0.6%, 95% CI -1.8%, 0.7%, p=0.38 in model 2), indicating potential residual confounding in the primary model. There was limited evidence for an association between partner smoking and hypomethylation at *AHRR* (cg05575921: 0.8%, 95% CI -1.7%, 0.1%, p=0.07 in model 1; −0.3%, 95% CI 1.0%, 0.4%, p = 0.46 in model 2 and -2.2%, 95% CI -5.0%, 0.5%, p = 0.12 in model 3), in contrast to the association observed in relation to own smoking (−3.7%, 95 % CI -4.6%, 2.8%, p=1.66 × 10^−14^), as shown in ***Supplementary File 4***.

We performed a search of the top CpG sites identified in the EWAS analysis in two publicly available repositories of published EWAS literature: the EWAS Catalog (MRC-IEU University of Bristol, 2018) and EWAS Atlas (BIG Data Center, 2019), as shown in ***Supplementary File 5***. 4 CpG sites (cg17672850; cg18866792; cg13249774; cg11549025) identified in relation to partner smoking were found to be related to other traits: colorectal laterally spreading tumour, adenoma, Down syndrome, atopy, immune system disease and myalgic encephalomyelitis (chronic fatigue syndrome). Just one CpG site (cg18866792) has been previously related to own smoking in a large EWAS meta-analysis (Joehanes et al., 2016). Of the 30 CpG sites identified at p<1x10-5 in relation to own smoking (model 4), 13 had been previously related to other traits: smoking, current versus never smoking, former versus never smoking, maternal smoking in pregnancy, serum cotinine, educational attainment and alcohol consumption per day. The 17 CpG sites related to own smoking which were not identified in the EWAS Catalog and EWAS Atlas, included sites located close to regions where DNA methylation changes have previously been identified in relation to smoke exposure, including *F2RL3* (cg21911711) as well as some novel gene regions, e.g. *SLAMF7* (cg00045592), *HEPACAM2* (cg04992673) and *PRDM2* (cg05297322).

**Fig. 3:**
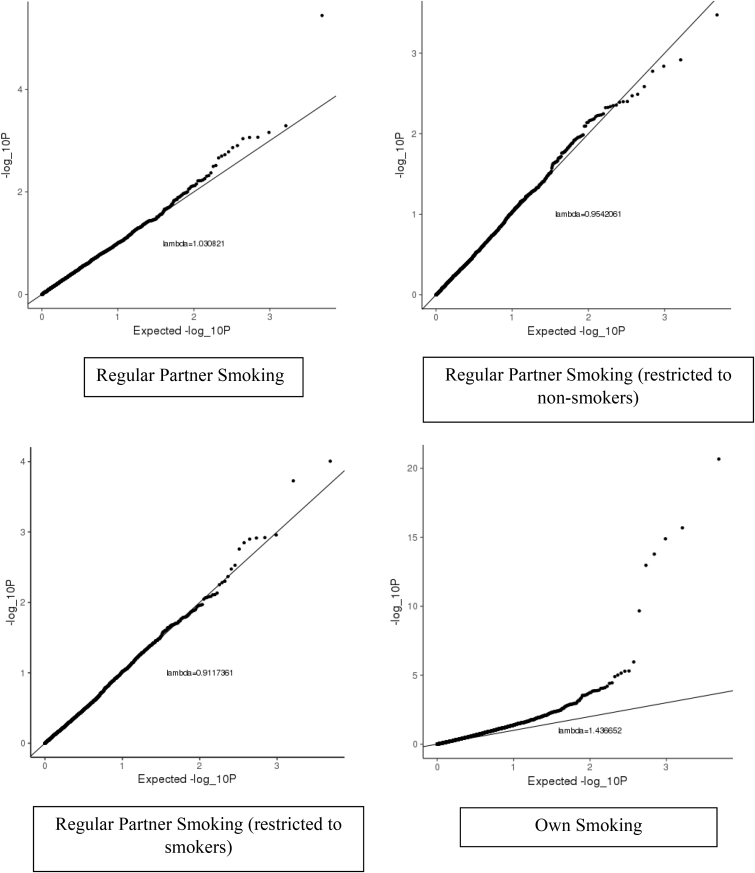
QQ plots and lambda values of DNA methylation at CpG sites previously associated with smoking in relation to partner and own smoking in Understanding Society.

QQ plots to assess the enrichment of CpG sites previously associated with smoke exposure in a large EWAS meta-analysis (Joehanes et al., 2016) in relation to partner and own smoking in *Understanding Society* are presented in Fig. 3. There was no clear enrichment of previous identified smoking-related signals in relation to partner smoking (lambdas 1.03, 0.95 and 0.91; Wilcoxon rank sum p-value p=0.34, p=0.87 and p=0.99 for models 1–3). Inflation of signals was observed in relation to own smoking (lambda 1.43; Wilcoxon rank sum p-value p<2.2 × 10^−16^ for Model 4).

## 4 Discussion

We conducted an analysis to investigate the impact of PS exposure, assessed in relation to partner's smoking status, on DNA methylation changes measured in peripheral blood. DNA methylation was not robustly related to partner's smoking status, with no CpG sites surpassing the epigenome-wide significance threshold (p<5.97 × 10^−8^). There was, however, suggestive evidence for an interaction between partner's and own smoking status on DNA methylation levels at a number of CpG sites, albeit not at epigenome-wide significance. This suggests that the impact of passive smoke exposure on the DNA methylation might be modified by whether the individual smokes themselves. Interestingly, there was limited overlap between those sites identified in relation to passive smoke exposure compared with those found in relation to a participant's own smoking.

Of the top CpG sites that were related to PS at a less stringent p-value threshold of p<1 × 10^−5^, cg18866792 (*MARCH4*) has previously been identified in relation to current versus never smoking in a large EWAS meta-analysis (Joehanes et al., 2016), although it was not related to own smoking in our study. However, it is important to recognise that the sample size of our study was significantly smaller than that in the EWAS meta-analysis; 769 versus 15,907 participants, respectively. *MARCH4* is a protein coding gene which is a member of the MARCH family of membrane bound E3 ubiquitin ligases. DNA methylation at this site has been previously identified in relation to Down syndrome and gestational age, as determined from our evaluation of the EWAS Catalog (MRC-IEU University of Bristol, 2018) and EWAS Atlas (BIG Data Center, 2019) resources. CpG site cg13249774 is annotated to the *LRP5* gene, which encodes the low-density lipoprotein receptor-related protein 5. DNA methylation at this site has previously been identified in relation to atopy, allergy and immune system disease.

In the EWAS of own smoking, we found some evidence for several CpG sites which have not been previously identified in large-scale EWAS, including cg21911711 annotated to *F2RL3*, which encodes the protein coagulation factor II (thrombin) receptor-like 3, and cg00045592 annotated to *SLAMF7*, a self-ligand receptor of the signalling lymphocytic activation module.

A small number of studies have previously looked at the impact of SHS on DNA methylation (Callahan et al., 2019; Reynolds et al., 2016; Wilhelm-Benartzi et al., 2011). Both Callahan et al. (2019) and Wilhelm-Benartzi et al., 2011 looked at SHS exposure and DNA methylation in cancerous tissues rather than peripheral blood, whereas Reynolds et al. (2017) assessed DNA methylation only at cg05575921 (*AHRR*). The relationship between cigarette smoking and *AHRR* methylation, a known tumour suppressor, has been well documented in previous studies (Monick et al., 2012; Tsai et al., 2018). Reynolds et al. (2017) concluded that there was an inverse association in non-smokers between the number of hours in close contact with people cigarette smoking indoors and DNA methylation at cg05575921 (*AHRR)* particularly with >10 h per week of SHS exposure (Reynolds et al., 2016). Whilst in our study we only found weak evidence for an association between partner smoking status and DNA methylation at cg05575921 (p=0.07), this could be because SHS exposure in our study was lower than in Reynolds et al. (2017). Of note, in this previous study there was limited evidence of association between SHS exposure <9 h per week and DNA methylation. However, residual confounding by own smoking may have biased the results of Reynolds et al. (2017).

There are several limitations to this study. While partners' smoking status was used as a proxy for PS exposure, the actual extent of exposure is dependent on several factors, including: the number of cigarettes smoked in the presence of other people, the proportion of smokers to non-smokers in the household, and the room ventilation. It was also assumed that the majority of PS exposure took place in the home, not accounting for PS exposure in the workplace or other indoor venues participants may frequent. In addition, we did not consider the impact of thirdhand smoke exposure from dermal absorption, ingestion and inhalation; further details are described elsewhere (Kuo and Rees, 2019). These data were not available for analysis. Furthermore, the impact of exposure measurement error must be considered, from self-reporting methods and reports of partner's smoking patterns.

While our EWAS models were adjusted for common covariates (age, sex, cell types and batch), as well as surrogate variable analysis in order to capture residual confounding in the models (Leek and Storey, 2007), there are plausible confounders which could have been specifically considered (e.g. alcohol consumption and educational attainment). This could explain why some of the CpG sites identified in relation to own smoking analysis have been previously related to alcohol and education in the literature. In addition, within the *Understanding Society* cohort, no data were collected on prenatal smoke exposure, which has been robustly related to long-term changes in DNA methylation of the offspring exposed (Richmond et al., 2018). While we must recognise the chance that some of the individuals exposed to PS were also prenatally exposed, which may have led to confounding by prenatal smoke exposure, none of the top CpG sites identified in relation to PS had been previously related to maternal smoking in the literature.

Participants with partners that smoked had a mean age of 56.1 years, whilst for participants with partners that do not smoke had a mean age of 54.8 years. As such, the findings from this study may not be particularly generalisable to the general population. However, as smoking is now at a much lower prevalence among younger people than it used to be; 16.8% of 18–24 year-olds were smoking in the UK and 19.2% of 25–34 year-olds (Office for National Statistics, 2018), this suggests that PS exposure via partners' smoking is likely to be less of a concern among younger populations than it used to be.

This study also has important strengths. The use of the Illumina Infinium EPIC array allowed us to assess DNA methylation in peripheral blood at over 850,000 CpG sites across the epigenome in relation to PS exposure, as assessed based on partners' smoking status, in a sample of 769 individuals. We were also able to account for participants' own reported smoking, through adjustment and stratification, in order to minimise the confounding effect of a participant's own smoke exposure on the association between PS and DNA methylation. This was evidenced by the limited overlap between DNA methylation signals found in relation to PS and own smoking.

Despite the relatively small sample size in this analysis, the evaluation of DNA methylation in relation to own smoking status indicates that we had adequate power to detect true methylation signals of the magnitude observed in relation to own smoking, suggesting that PS exposure has much less of an impact on DNA methylation compared with own smoking (as *a priori* hypothesized). This is in line with what we know about exposure levels and health effects of smoking and PS (Öberg et al., 2011).

## 5 Conclusion

The results of this study indicate that PS exposure in households does not have a strong effect on the DNA methylation. In particular, the epigenetic signature associated with PS exposure is much less pronounced than that of own smoking. While the impact of PS exposure on adult and child health is well known, it is unlikely that changes to DNA methylation play an important role in the role in the development of these health effects.

## Credit author statement

Paige Hulls: Formal analysis; Roles/Writing – original draft, Frank de Vocht: Supervision; Writing - review & editing, Yanchun Bao: Resources; Writing - review & editing, Caroline Relton: Conceptualization; Writing - review & editing, Richard Martin: Supervision, Writing - review & editing, Rebecca Richmond: Conceptualization; Formal analysis; Roles/Writing – original draft; SupervisionRCR and CLR contributed to the conception of the study. PMH and RCR conducted the analysis and wrote the first draft of the manuscript. RCR, FdV and RMM contributed to supervision of the study. YB provided access to the relevant datasets in *Understanding Society*. FdV, RMM, YB and CLR critically commented on the first draft of the manuscript.

### Funding

PMH is funded by a 10.13039/100010269Wellcome Trust 4-year studentship (108902/Z/15/Z). RCR is a de Pass VC research fellow at the University of Bristol. RCR, RMM and CLR are members of the 10.13039/501100000265MRC Integrative Epidemiology Unit at the University of Bristol funded by the 10.13039/501100000265Medical Research Council (MC_UU_00011/5). The work was also supported by 10.13039/501100000289Cancer Research UK (C18281/A19169) and the 10.13039/501100000269Economic and Social Research Council (10.13039/501100000269ESRC) (ES/N000498/1). RMM is supported by the 10.13039/501100000272National Institute for Health Research (10.13039/501100000272NIHR) 10.13039/100015250Bristol Biomedical Research Centre which is funded by the 10.13039/501100000272National Institute for Health Research (10.13039/501100000272NIHR) and is a partnership between 10.13039/100012141University Hospitals Bristol NHS Foundation Trust and the University of Bristol. Department of Health and Social Care disclaimer: The views expressed are those of the authors and not necessarily those of the NHS, the NIHR or the Department of Health and Social Care. YB is supported by the 10.13039/501100000269ESRC (ES/M008592/1). FdV is supported by the 10.13039/501100012349NIHR School for Public Health Research and 10.13039/501100000272NIHR Applied Research Collaboration West. The epigenetics methylation data were analysed by the University of Exeter Medical School (10.13039/501100000265MRC grant K013807) and further facilitated by the University of Essex School of Biological Sciences.

The funders had no role in study design, data collection and analysis, decision to publish or preparation of the manuscript. This publication is the work of the authors and RCR will serve as a guarantor for the contents of this paper.

### Ethics approval and consent to participate

Data governance was provided by the METADAC data access committee, funded by 10.13039/501100000269ESRC, Wellcome, and 10.13039/501100000265MRC (2015–2018: Grant Number MR/N01104X/1 2018–2020: Grant Number ES/S008349/1).Ethical approval for 10.13039/100012232Understanding Society was obtained from the National Research Service (10.13039/100012232Understanding Society – UK Household Longitudinal Study: A Biosocial Component, Oxfordshire A REC, Reference: 10/H0604/2). All our consents can be found here: https://www.understandingsociety.ac.uk/documentation/health-assessment/fieldwork-documents.

## Declaration of competing interest

The authors declare that they have no known competing financial interests or personal relationships that could have appeared to influence the work reported in this paper.
