# Supplementary figures and images for: DNA methylation signature of passive smoke exposure is less pronounced than active smoking: The Understanding Society study
Source: Environ Res. 2020 Nov;190:109971. doi: 10.1016/j.envres.2020.109971 (PMC7536273; doi:10.1016/j.envres.2020.109971)

# Singular Value Decomposition Analysis (SVD)

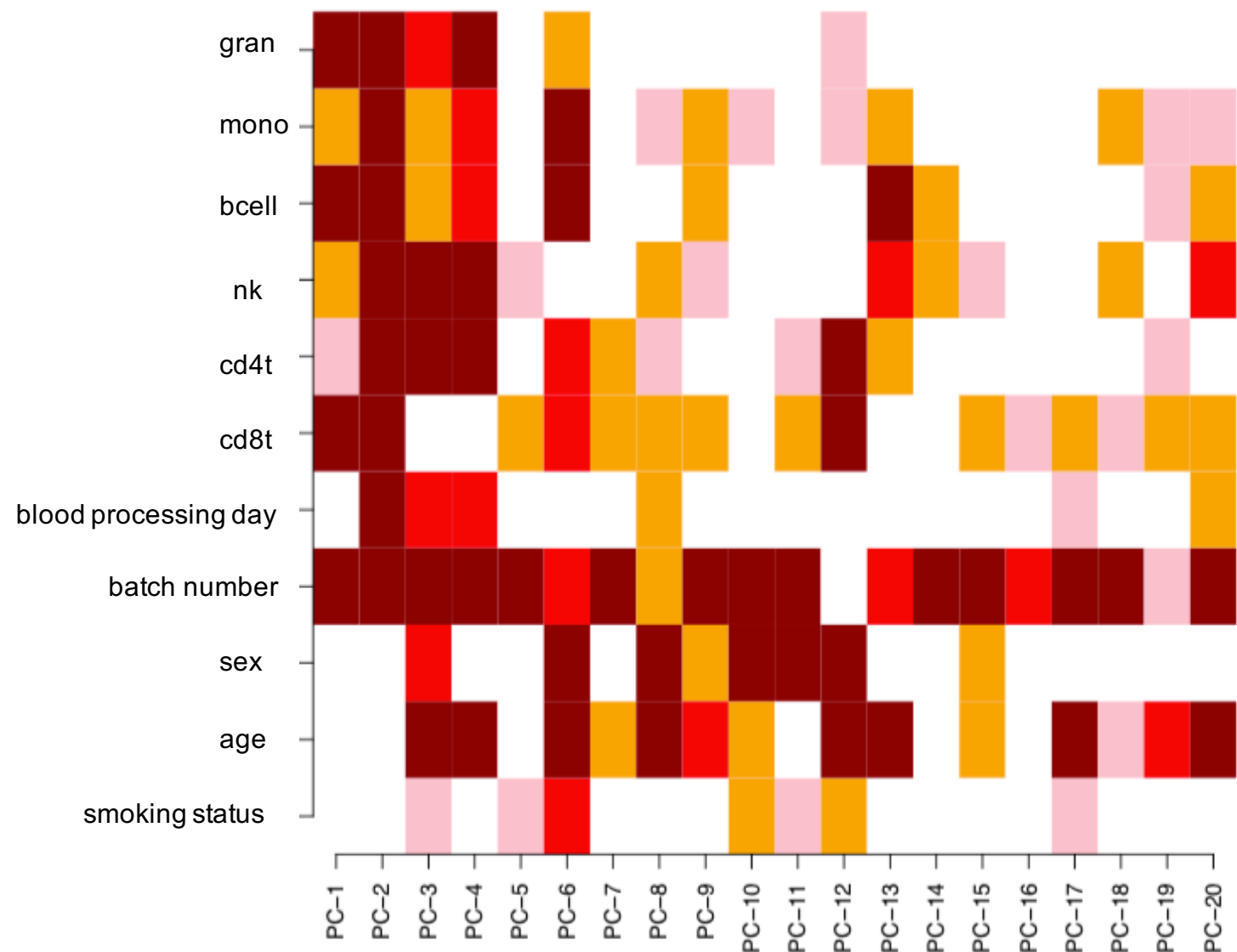

Supplement: Multimedia component 1 [file mmc1.pdf]
