# Supplementary material for: DNA methylation signature of passive smoke exposure is less pronounced than active smoking: The Understanding Society study
Source: Environ Res. 2020 Nov;190:109971. doi: 10.1016/j.envres.2020.109971 (PMC7536273; doi:10.1016/j.envres.2020.109971)
Supplement: Multimedia component 3 [file mmc3.pptx]

## Slide 1
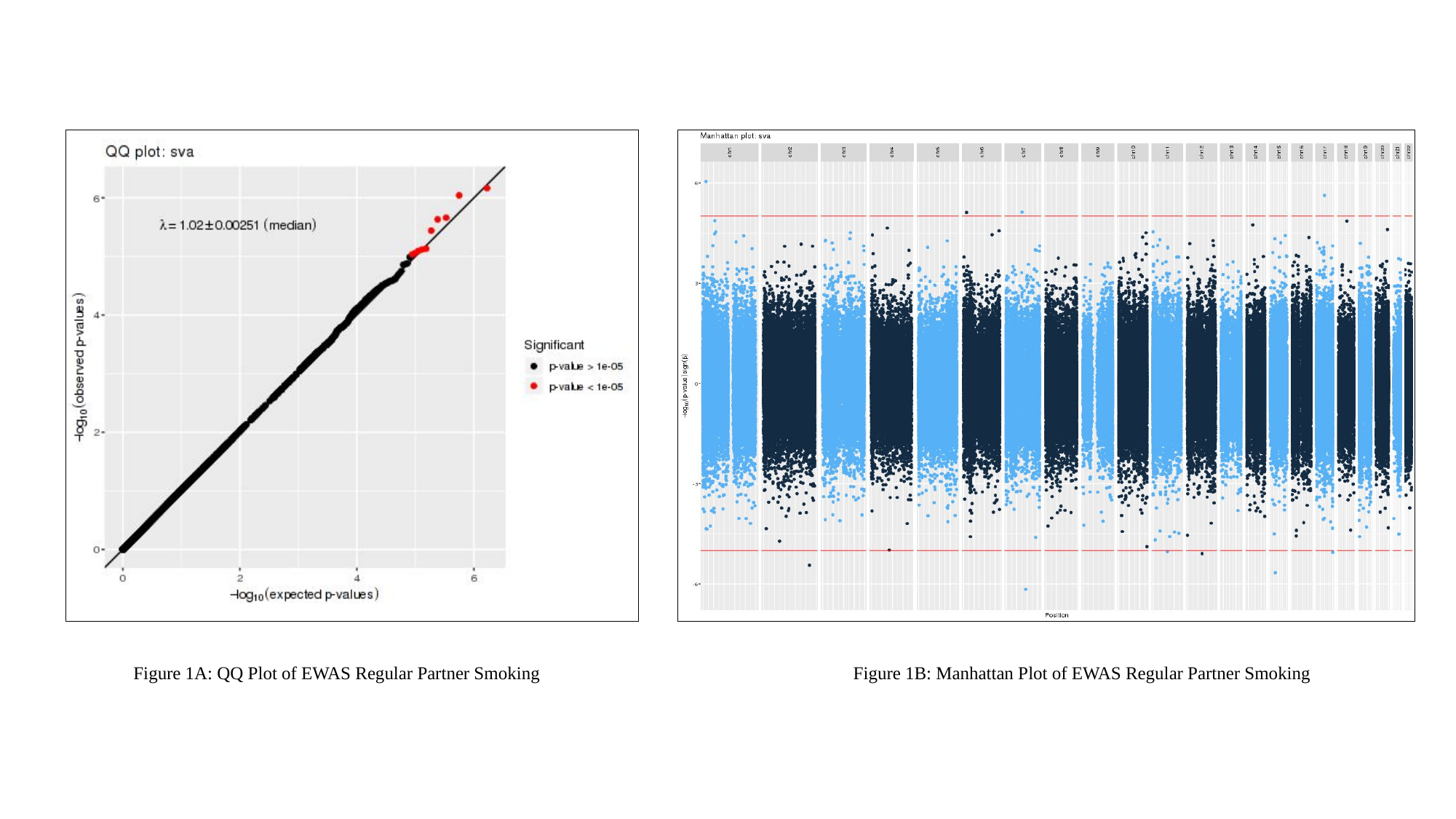

Figure 1B: Manhattan Plot of EWAS Regular Partner Smoking
Figure 1A: QQ Plot of EWAS Regular Partner Smoking

## Slide 2
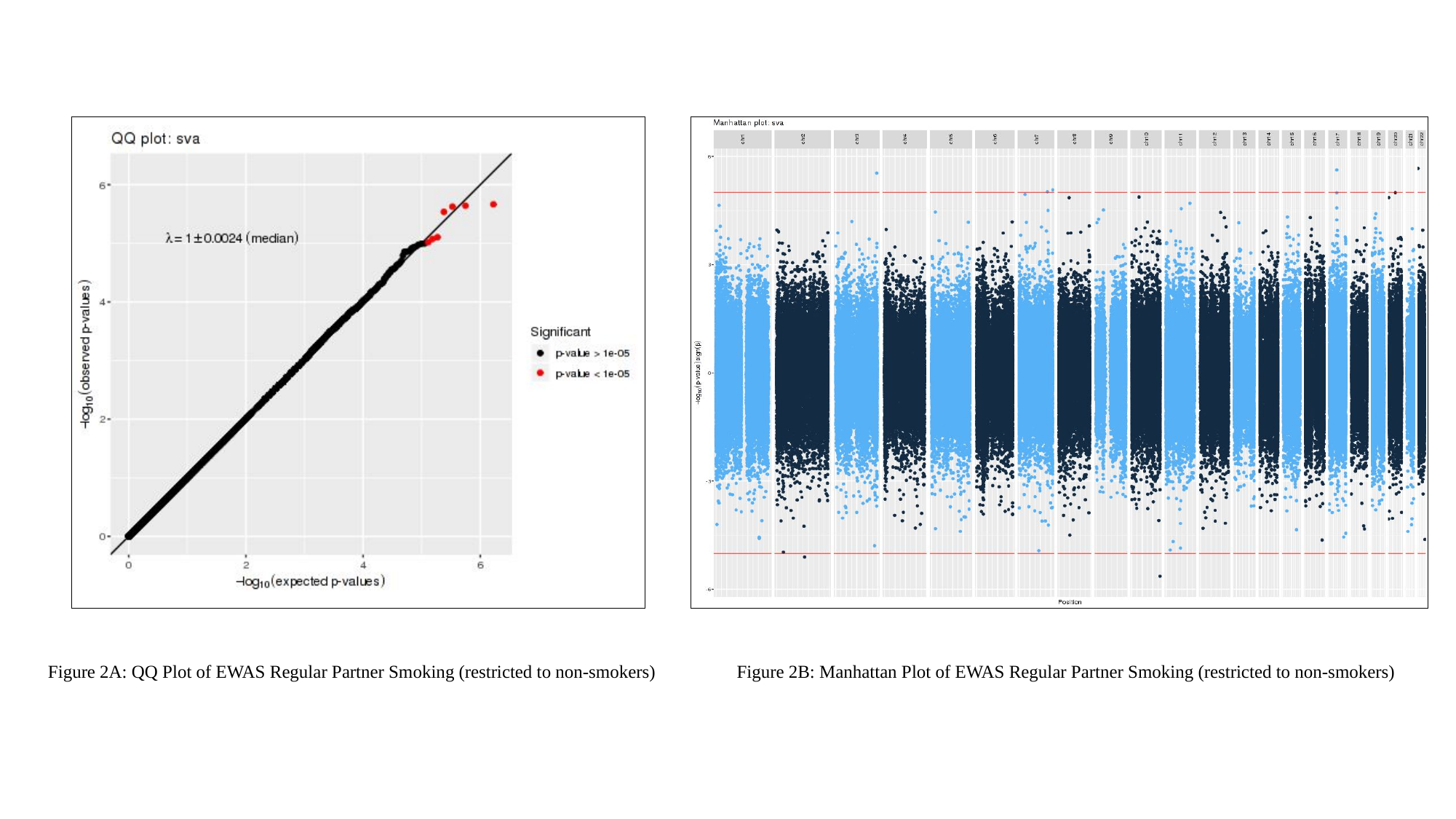

Figure 2A: QQ Plot of EWAS Regular Partner Smoking (restricted to non-smokers)
Figure 2B: Manhattan Plot of EWAS Regular Partner Smoking (restricted to non-smokers)

## Slide 3
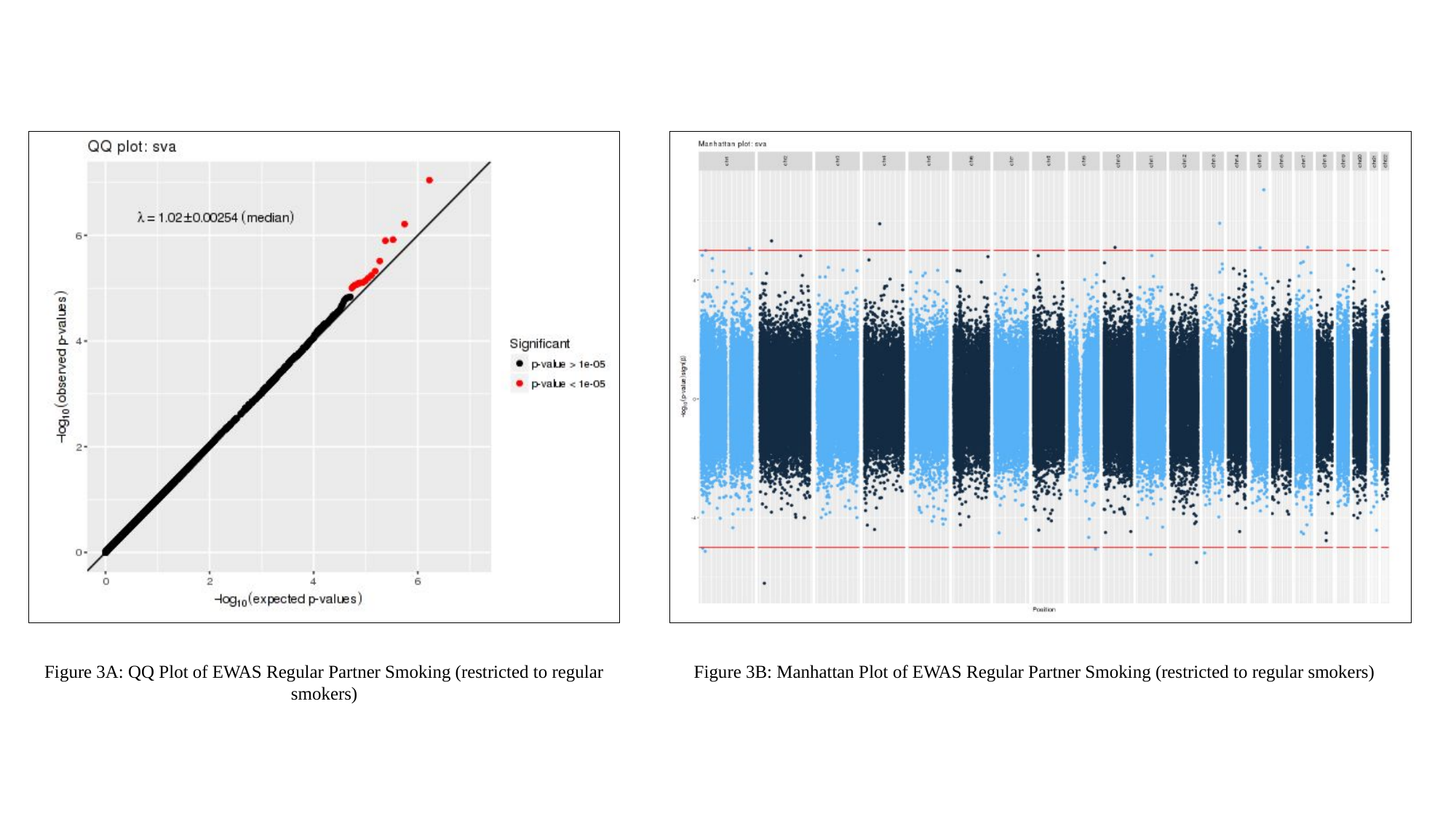

Figure 3A: QQ Plot of EWAS Regular Partner Smoking (restricted to regular smokers)
Figure 3B: Manhattan Plot of EWAS Regular Partner Smoking (restricted to regular smokers)

## Slide 4
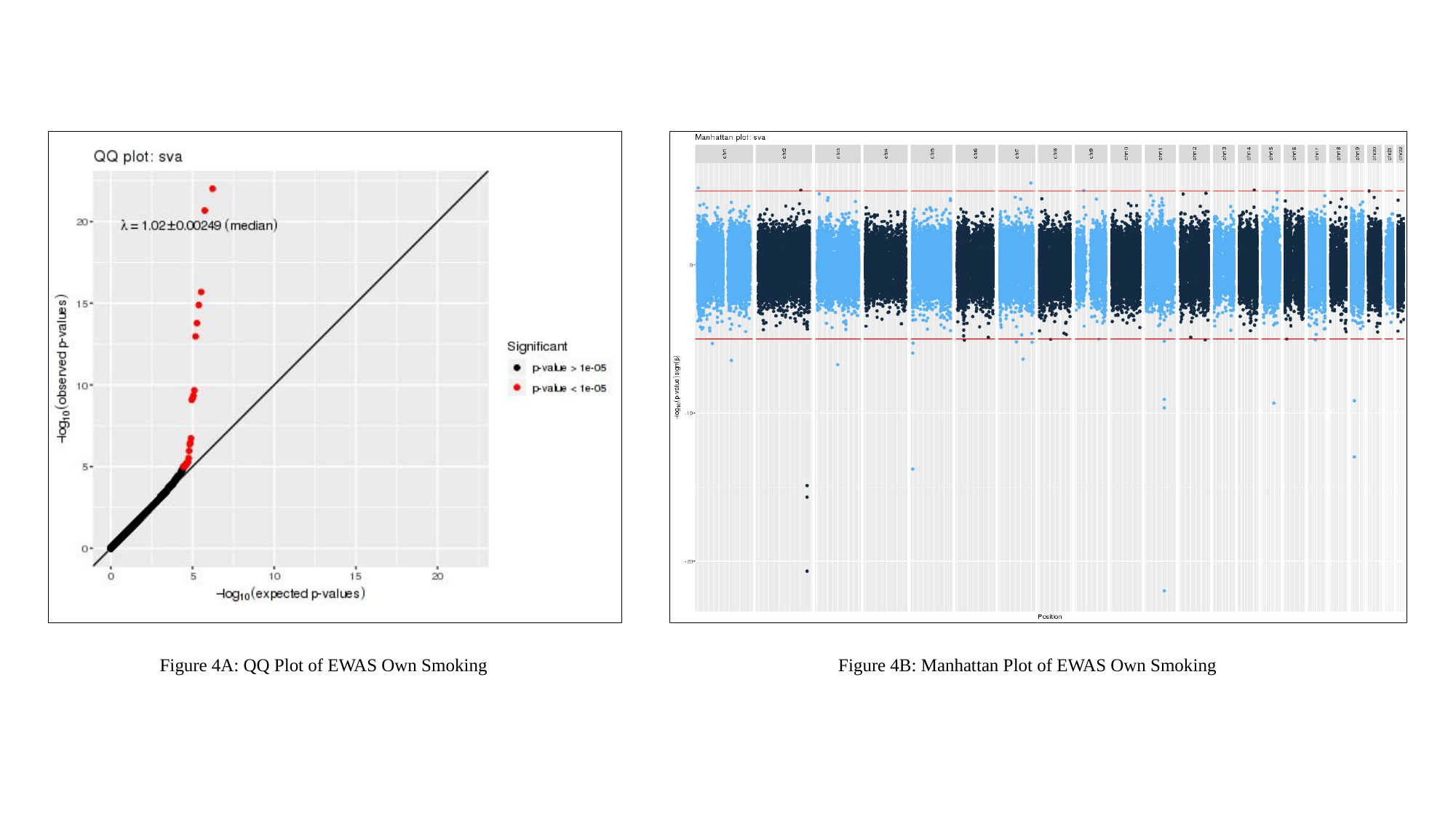

Figure 4A: QQ Plot of EWAS Own Smoking
Figure 4B: Manhattan Plot of EWAS Own Smoking
